# Hyperglycaemic effect of *Artocarpus communis* Forst (Moraceae) root bark aqueous extract in Wistar rats

**Published:** 2007

**Authors:** Stephen O Adewole, John AO Ojewole

**Affiliations:** Department of Anatomy and Cell Biology, Faculty of Basic Medical Sciences, College of Health Sciences, Obafemi Awolowo University, Ile-Ife, Osun State, Nigeria; Department of Pharmacology, Faculty of Health Sciences, University of KwaZulu-Natal, Durban

## Abstract

Decoctions and infusions of *Artocarpus communis* (Forst) (family: Moraceae) root bark are traditionally used among the Yoruba-speaking people of western Nigeria as folk remedies for the management, control and treatment of an array of human diseases, including type 2 diabetes mellitus. Although numerous bioactive prenylflavonoids have been isolated from the roots, stem bark and leaves of *A communis*, to the best of our knowledge, the effects of the plant’s root bark extract on animal models of diabetes mellitus have hitherto not been reported in the biomedical literature. In our pilot study, we observed that *A communis* root bark aqueous extract (ACE) raised blood glucose concentrations in rats. In view of this finding, the present study was undertaken to investigate the glycaemic effect of ACE in comparison with that of streptozotocin (STZ) in Wistar rats.

Four groups (A, B, C and D) of Wistar rats, each group consisting of 10 rats, were used in this study. Group A rats received distilled water in quantities equivalent to the volume of ACE administered. Diabetes mellitus was induced in the animals in groups B and C by intraperitoneal (ip) injections of STZ (75 mg/kg body weight). The rats in group C were additionally treated with ACE (50 mg/kg body weight ip) from the third to the tenth day following STZ treatment. Group D rats received ACE (12.5–100 mg/kg body weight ip) only.

The effects of ACE were compared with those of STZ on blood glucose concentrations, serum and pancreatic insulin levels, hepatic hexokinase (HXK) and glucokinase (GCK) activities, and hepatic glycogen contents in the experimental animal paradigm used. The rats in treated groups B, C and D exhibited pronounced polyuria, hypo-insulinaemia and hyperglycaemia. Group D rats developed significant hyperglycaemia (*p* < 0.05) immediately after ACE administration, whereas groups B and C rats became hyperglycaemic 24 to 72 hours post STZ and STZ 1 ACE treatments, when compared with the control group A rats. Hepatic glycogen contents significantly increased (*p* < 0.05), while HXK and GCK activities significantly decreased (*p* < 0.05) in the treated groups B, C and D rats, when compared with the control group A rats.

The findings of this laboratory animal study indicate that A communis root bark aqueous extract induced acute hyperglycaemia in Wistar rats, and that it disrupted the biochemical variables of the rat pancreas and liver.

## Summary

In our search for plants with the potential for use as effective and safe ethnomedical remedies in the management of a catalogue of human diseases, we have subjected many African medicinal plants to phytochemical and pharmacological investigations in our laboratories. We have shown that some African medicinal plants possess hypoglycaemic, anti-inflammatory, analgesic, and other pharmacological properties.^1^,^2^ One of the African medicinal plants that we recently subjected to pharmacological investigation is *Artocarpus communis*.

*A communis* (Forst) is a perennial, evergreen, terrestrial, single-stemmed, erect flowering plant, popularly known in English as breadfruit tree because of the bread-like texture of its edible fruits. It is a member of the Moraceae family which consists of about 50 genera and over 1 000 species.^3^ The breadfruit tree is a fast-growing plant of up to 20–30 m in height, and with a trunk of up to 1–2 m in diameter. All the morphological parts of the tree, including the unripe fruit, are rich in milky, gummy latex.

The shoots, bark and latex of the plant have been reported to have many traditional, ethnomedical uses. For example, in the West Indies, a decoction of the leaves is used to lower elevated blood pressure and to relieve asthma. In Taiwan, the leaves are used for fever and liver disorders, while the sap is used for thrush, stomach pain and dysentery. Root extracts are used as an antibacterial remedy against gram-positive bacteria, and as anticarcinogenic agents.^4^ In Nigeria, the root bark is traditionally used for a variety of human ailments, including management or treatment of diarrhoea, dysentery and type 2 diabetes mellitus.

Natural compounds isolated from *A communis* roots, stem bark and leaves include the prenylflavonoids: dihydrocycloartomunin, dihydroisocyloartomunin, heteroflavanones, cylomorusin, artonins A and B, cycloheterhyllin, cyclocomunin, cycloartomunin artocarpanone A, and cudraflavone;^5^,^6^ and the flavonoids: dihydroartomunoxanthone, artomunoisoxanthone, cyclocomunomethonol and artomunoflavanone.^7^ Some of these compounds are known to possess significant antiplatelet^8^ and anti-inflammatory^9^,^10^ activities.

Generally, flavonoids constitute a group of naturally occurring compounds widely distributed as secondary metabolites in the plant kingdom. They have been recognised for having interesting pharmacological properties, such as anti-inflammatory, anti-allergic, antiviral, antibacterial and antitumour activities.^11^ One of these flavonoids, quercetin (3,5,7,3',4'-pentahydroxyflavone) prevents oxidative injury and cell death by several mechanisms, including scavenging oxygen radicals,^12^ protecting cells and tissues against lipid peroxidation, and chelating metal ions.^13^

Diabetes mellitus is probably the single most important metabolic disease that is widely recognised as one of the leading causes of death and disability in the world today. Experimentally, it is known that streptozotocin (STZ)-induced diabetes mellitus causes functional and structural changes in some tissues and organs of animals. Experimental evidence has also demonstrated that some of the toxic, deleterious effects of STZ are attributable to induction of metabolic abnormalities, which lead to an increase in the generation of reactive oxygen species (ROS).^14^ Apart from the production of ROS, STZ also inhibits free radical scavenger enzymes.^15^ The superoxide radical has been implicated in lipid peroxidation, DNA damage and sulphydryl oxidation.^16^,^17^ However, the mechanism of the cytotoxic action of STZ is not fully understood.

The present study was prompted by the claim of some traditional health practitioners in western Nigeria that decoctions and infusions of *A communis* root bark are effective remedies for the management and control of type 2 adult-onset diabetes mellitus. The aims of the present study were, therefore, to examine (1) the glycaemic effect of *A communis* root bark aqueous extract in Wistar rats, and (2) the effects of the extract on the biochemical variables of the rat pancreas and liver.

## Materials and methods

The experimental protocol and procedures used in this study were approved by the Ethics Committee of the University of KwaZulu-Natal, Durban and conform with the *Guide to the Care and Use of Animals in Research and Teaching.*^18^

## Animals

This study was carried out in healthy male and female Balb C mice (*Mus domesticus*) weighing 20 to 25 g, and healthy young adult Wistar rats (*Rattus norvegicus*) of both sexes weighing 250 to 300 g. The animals were housed under standard laboratory conditions of light, temperature and humidity and were given standard rat pellets and tap water *ad libitum*. The rats were randomly divided into four experimental groups: group A (distilled water-treated control), group B (STZ treated), group C (STZ + *A communis* root bark extract treated), and group D (root bark extract treated) rats.

Each group consisted of 10 rats. All the animals were fasted for 16 hours, but still allowed free access to water before the commencement of our experiments. The mice were used for acute toxicity testing of the crude plant extract, while the rats were used for glycaemic evaluations of the extract.

## Plant material

Pieces of the root bark of *A communis* were collected in Ile-Ife, western Nigeria, between April and May 2006 and identified by the taxonomist/curator of the Department of Botany, Obafemi Awolowo University, Ile-Ife, Nigeria. A voucher specimen of the plant has been deposited in the Herbarium of the University’s Botany Department.

Pieces of *A communis* fresh root bark were air-dried at room temperature and 1 kg was milled to a fine powder in a Waring commercial blender. The powdered root bark was macerated in distilled water and extracted twice, on each occasion with 2.5 l of distilled water at room temperature for 48 h. The combined aqueous extracts were concentrated to dryness under reduced pressure at 60 ± 1°C in a rotary evaporator. The resulting extract was freeze-dried, finally giving 56.23 g (5.62% yield) of a clay-coloured, powdery, crude aqueous root bark extract of *A communis* (ACE). Without any further purification, aliquot portions of the crude residue were weighed and dissolved in distilled water for use on each day of our experiment.

## Acute toxicity testing

The median lethal dose (LD_50_) of *A communis* root bark aqueous extract was determined in mice using a modified method of Lorke.^19^ Mice fasted for 16 h were randomly divided into groups of 10 mice each. Graded doses of ACE (12.5, 25, 50, 100, 200, 400, 800 and 1 600 mg/kg) were separately administered intraperitoneally to the mice in each of the test groups. Each of the mice in the control group was treated with distilled water (3 ml/kg ip) only. The mice in both the test and control groups were then allowed free access to food and water, and observed over a period of 48 h for signs of acute toxicity. The number of deaths produced by the extract within this period of time was noted and recorded. Log dose–response plots were constructed for the plant extract, from which the LD_50_ value of the plant extract was determined.

## Induction of experimental diabetes mellitus

Diabetes mellitus was induced in groups B and C test rats by intraperitoneal injections of STZ (75 mg/kg), freshly dissolved in 0.1 mmol/l citrate buffer.^20^ Control rats were injected with volumes of distilled water equivalent to the volume of administered ACE. The test animals in groups B and C became diabetic within 48 hours after STZ administration. Diabetes was allowed to develop and stabilise in these STZ-treated rats over a period of 3–7 days. Group C rats additionally received intraperitoneal injections of ACE (50 mg/kg) daily from the third to the tenth day after STZ treatment.

All the animals in groups A, B, C and D were maintained under laboratory conditions of light, humidity and temperature. Before the commencement of our experiments, both the control normoglycaemic and STZ-treated diabetic (hyperglycaemic) test rats were fasted for 16 h, but still allowed free access to water throughout. At the end of the 16-h fasting period, taken as 0 time, blood glucose levels (initial glycaemia, G_0_) of the fasted normal and STZ-treated diabetic rats were determined and recorded.

## Evaluation of hyperglycaemic property of ACE

The test compound (ACE, 12.5–100 mg/kg) was administered to group D rats. Blood glucose concentrations (Gt) were again determined and recorded 15 min, 30 min, one, two, four, eight, 24 and 48 hours following ACE administration to the animals. Each rat was restrained in a cage and blood samples were collected from the tail tip vein for blood glucose analysis.

## Blood glucose and serum insulin estimations

Blood samples were taken one day before STZ treatment, and also on various days after induction of diabetes mellitus. Blood glucose concentrations were determined by means of Bayer’s Elite® Glucometer and compatible blood glucose test strips.^21^ Fasted STZ-treated rats with blood glucose concentrations ≥ 18 mmol/l were considered to be diabetic and used in this study. Serum insulin concentrations were determined by an enzyme-linked immunosorbent assay (ELISA), using a commercial kit (Crystal Chem, Chicago, IL, USA).

## Pancreatic insulin determination

The splenic regions of pancreatic tissues were removed from euthanised rats, weighed and homogenised on various experimental days, in acid−ethanol solution (75% ethanol, 23.5% distilled water, 1.5% concentrated HCl). After overnight incubation at 4°C, the suspensions were centrifuged and the supernatants collected and assayed for insulin content using a competitive ELISA kit.^22^ Plates were coated with rabbit anti-guinea pig Ig secondary Ab (Organon Teknoka, Durhan, NC), followed by incubation with a guinea pig anti-human insulin Ab (Cortex Biochem, San Leandro, CA).

Following two washing steps, various extract dilutions or insulin standards (Linco Research, St. Louis, MO) were mixed with a constant concentration of HRP-conjugated rat insulin (Organon Teknika) for four hours at room temperature, or at 4°C overnight, before competitive capturing was allowed for three hours. After washing five times, Sigma FAST OPD tablets (Sigma, St. Louis, MO) were used as substrate. Results were analysed using Ceres 900 C ELISA reader with KC3 software (Bio-Tek Instruments, Winooski, VT). The pancreatic extract total protein was measured by a protein assay (Bio-Rad, Richmond, VA) according to the manufacturer’s recommendations using BSA standards (Bio-Rad).

## Determination of HXK and GCK activities

Frozen liver tissues (1 g) were homogenised at 4°C in 9 ml of cold buffer solution (pH 7.4) containing Na-HEPES (50 mM), KCl (100 mM), EDTA (1 mM), MgCl_2_ (5 mM) and dithiothreitol (DTE) (2.5 mM), using a glass-Teflon Potter homogenizer. The suspension formed was centrifuged at 12 000 3 g for 1 h at 4°C. The clear supernatant was used for the measurement of HXK and GCK activities by the coupled enzyme assay procedure of Davidson and Arion.^23^

The incubation mixture contained the following ingredients in a final volume of 1 ml: HEPES (50 μmol), KCl (100 µmol), MgCl_2_ (7.5 μmol) and DTE (2.5 μmol); fatty acid-free bovine serum albumin (10 mg), NAD^+^ (0.5 μmol), G-6-PD (4 U), liver supernatant (100 μl) for HXK assay, or 10 μl for total HXK and GCK assays; and D-glucose (0.5 μmol) for HXK and 10 μmol for total enzyme activities. Both the control and test tubes were pre-incubated at 25°C for 5 min. Distilled water (0.2 ml) was added to the control tubes, and to start the reactions in the test tubes, 0.2 ml of a solution containing 0.5 μmol ATP was added.

Control tubes were adjusted to zero absorbance in a DU-7 spectrophotometer at 340 nm wavelength, and the increase in absorbance in the test tubes at this wavelength was plotted against the time period of 15 min. Total enzyme activities (GCK 1 HXK and HXK activities) were calculated in terms of mU/ml of the liver supernatant. One milliunit of the enzyme corresponds to the amount of the enzyme producing 1 nmol of NADH per min under assay conditions at 25°C. Hexokinase activities were subtracted from the total HXK 1 GCK activities to obtain glucokinase activities. Protein content of the liver homogenates was determined using bicinchoninic acid (BCA) protein assay reagent (Pierce Chemical Company, Rockford, Chicago, USA).^24^

## Liver glycogen assay

Glycogen levels in the liver extracts were determined as described by Chung and Dao.^25^ Pieces of liver tissues were removed immediately after euthanisation, rinsed with cold saline and weighed. Thereafter, a small piece of the liver tissue was isolated and frozen in liquid nitrogen. Liver tissue extract (0.1 g) was dissolved in 30% KOH and heated at 100°C for 10 min, followed by 3-min room temperature incubation. The sample was diluted (1:10) with KOH and vortexed. Anhydrous ethanol (1.1−1.2 volumes) was added to precipitate glycogen from alkaline digestate, and the samples were centrifuged at 5 700 rpm for 15 min. The supernatant was carefully removed and the pellet was resuspended in 0.5 ml distilled water. One millilitre of a 0.2% anthrone reagent (0.2 g in 100 ml of 98% H_2_SO_4_) was quickly added and mixed, and the mixture was incubated at room temperature for 30 min. The samples were then measured at 620 nm with a spectrophotometer.

## Statistical analysis

Data obtained were expressed as means (± SEM) and analysed using repeated measures of variance. The differences between the means were analysed statistically with one-way analysis of variance (ANOVA; 95% confidence interval). Values of *p* < 0.05 were taken to imply statistical significance.

## Results

## Acute toxicity testing

Intraperitoneal administrations of relatively low doses of ACE (1–50 mg/kg) were found to be safe in mice, however, moderate to high doses (> 100 mg/kg ip) were toxic or lethal to them. The LD_50_ value of the plant extract was found to be 130 ± 12 mg/kg in mice, suggesting that *A communis* root bark aqueous extract is toxic to mice.

## Blood glucose, insulin levels and body weights

**Fig. 1.**
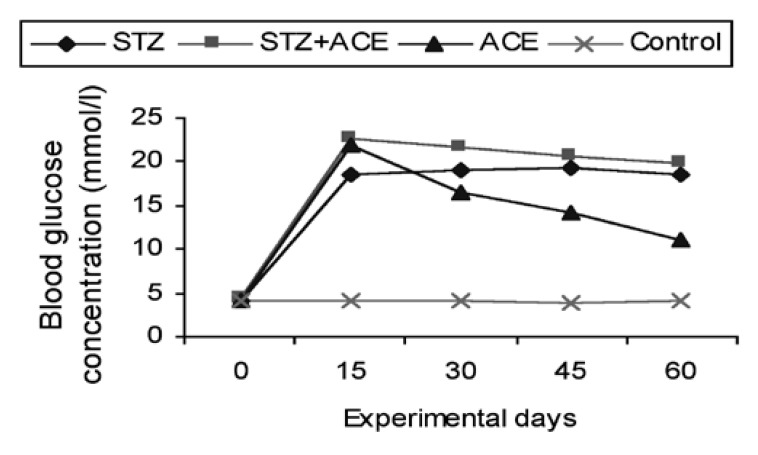
Blood glucose concentrations of STZ-, STZ + ACE-, and ACE-treated rats. The figure illustrates a persistent hyperglycaemic state in all three treated groups. There was a marked reduction in the blood glucose levels of the ACE-treated group of rats from the third week of treatment, but this reduction did not reach the normoglycaemic state of the control rats throughout the study period. Results are expressed as means of eight to 10 observations.

Changes in blood glucose and serum insulin levels and changes in body and pancreatic weights of the four animal groups studied are shown in Tables 1, 22 and 3. The three treated rat groups, B (STZ treated), C (STZ 1 ACE treated) and D (ACE treated) showed significant increases (*p* < 0.05) in blood glucose levels when compared with those of the control group (Table 3). After three weeks, the blood glucose concentrations of the ACE-treated rats started to drop, but the reduction in the blood glucose levels never attained group A’s normoglycaemic levels throughout the study period (Table 3, Fig. 1). There was a progressive decrease in the body and pancreatic weights, and an increase in liver weight of the rats in groups B, C and D, whereas the body weights of the control group showed moderate but insignificant (*p* > 0.05) increases (Table 1).

**Table 1:** Changes In Body, Pancreas And Liver Weights Of Control , STZ-, STZ + ACE-, And Ace-Treated Rats

	*Control*	*STZ-treated*	*STZ + ACE-treated*	*ACE-treated*
Body weights (g)	252 ± 10	217 ± 14^a^	206 ± 06^a^	212 ± 11^a^
Pancreatic weights (g)	1.08 ± 0.8	0.92 ± 0.5^b^	0.73 ± 0.4^b^	0.83 ± 0.2^b^
Liver weights (g)	7.79 ± 0.2	8.27 ± 0.5^b^	8.56 ± 0.4^c^	9.46 ± 0.8^c^

^a,b,c^Significant difference (*p* < 0.05) in the same row between various treatments and control group A rats.

**Table 2:** Effects Of Ace (12.5−100 MG/KG) On Blood Glucose Concentrations (MMOL/L) Of Wistar Rats

*ACE*	*Control*	*Treated*						
*(mg/kg)*	*0*	*30 min*	*1-h*	*2-h*	*4-h*	*8-h*	*24-h*	*48-h*
12.5	4.1 ± 0.5	14.6 ± 0.2^a^	15.2 ± 0.3^a^	17.3 ± 0.4^a^	18.5 ± 0.2^a^	18.3 ± 0.4^a^	17.7 ± 0.2^a^	10.5 ± 0.1^a^
25	4.0 ± 0.2	16.6 ± 0.5^b^	17.3 ± 0.2^b^	19.5 ± 0.3^b^	19.9 ± 0.4^b^	20.7 ± 0.5^b^	20.7 ± 0.1^b^	10.6 ± 0.5^b^
50	3.9 ± 0.3	18.0 ± 0.4^c^	18.6 ± 0.2^c^	20.2 ± 0.1^c^	21.7 ± 0.5^c^	21.7 ± 0.2^c^	22.1 ± 0.1^c^	10.4 ± 0.3^c^
100	4.2 ± 0.3	18.4 ± 0.5^d^	18.9 ± 0.7^d^	20.2 ± 0.4^d^	22.0 ± 0.3^d^	21.9 ± 0.6^d^	3.4 ± 0.5^d^	10.0 ± 0.8^d^

Values are expressed as means (± SEM) of eight to 10 rats for all groups. ^a,b,c,d^Significant difference (*p* < 0.05) in the same row between various treatments and control group A rats.

**Table 3:** Changes In Blood Glucose Concentrations Of Control , STZ-, STZ + ACE, And Ace-Treated Rats On Different Days Of The Study Period

*Parameters*	*Control*	*Treated*			
*Days/treatments*	*0*	*15*	*30*	*45*	*60*
Blood glucose concentrations (mmol/l)					
STZ treated	4.1 ± 0.5	18.6 ± 0.2^a^	19.2 ± 0.3^a^	19.3 ± 0.4^a^	18.5 ± 0.7^a^
STZ + ACE treated	4.3 ± 0.2	22.6 ± 0.5^b^	21.6 ± 0.2^b^	20.5 ± 0.3^b^	19.9 ± 0.4^b^
ACE treated	4.2 ± 0.3	21.8 ± 0.4^c^	16.6 ± 0.2^c^	14.2 ± 0.1^c^	10.7 ± 0.5

Values are expressed as means (± SEM) of eight to 10 rats for all groups. ^a,b,c^ Significant difference (*p* < 0.05) in the same row between various treatments and control group A rats.

## Serum and pancreatic insulin levels

**Fig. 2.**
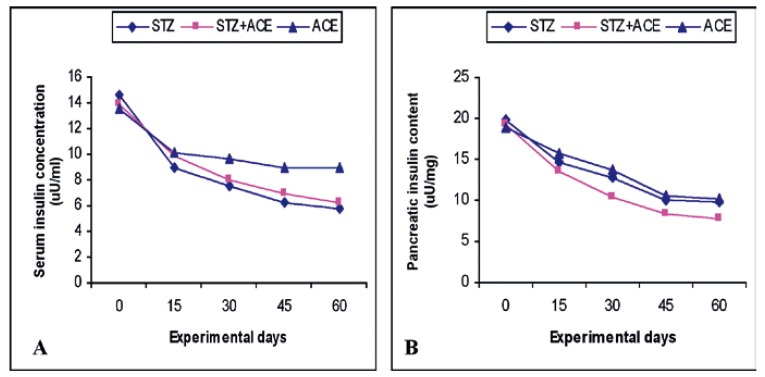
(A) Serum insulin and (B) pancreatic insulin levels of STZ, STZ + ACE- and ACE-treated rats. The figure illustrates the toxic, destructive effects of ACE on availability of insulin in the serum as well as on residual insulin levels of the pancreas. Results are expressed as means of eight to 10 observations.

The results summarised in Table 4A indicate that the serum insulin levels of the rats in the three treated groups B, C and D significantly decreased (*p* < 0.05) (Fig. 2) when compared with those of the control group. Furthermore, the pancreatic insulin levels of the treated rats significantly decreased (*p* < 0.05) (Fig. 2) when compared with those of the control rats (Table 4B). Values for the control rats were not significantly different.

**Table 4:** Effects Of STZ-, STZ + ACE-, And ACE-Induced Diabetes On (A) Serum Insulin And (B) Pancreatic Insulin Levels

*Parameters*	*Control*	*Treated*			
*days/treatment*	*0*	*15*	*30*	*45*	*60*
A. Serum insulin concentrations (μU/ml)					
STZ treated	14.1 ± 1.2	8.9 ± 1.6^a^	7.5 ± 1.3^a^	6.2 ± 1.7^a^	5.8 ± 1.4^a^
STZ + ACE treated	13.9 ± 1.5	9.9 ± 1.8^b^	8.0 ± 1.7^b^	6.9 ± 1.0^b^	6.2 ± 1.3^b^
ACE treated	13.5 ± 1.3	10.1 ± 1.5^c^	9.7 ± 1.2^c^	9.0 ± 1.7^c^	8.9 ± 1.4^c^
B. Pancreatic insulin concentrations (μU/mg protein)					
STZ treated	STZ treated	STZ treated	12.82 ± 1.7^a^	9.93 ± 3.5^a^	9.90 ± 2.7^a^
STZ + ACE treated	19.26 ± 2.3	13.47 ± 3.7^b^	10.29 ± 4.2^b^	8.29 ± 1.1^b^	7.72 ± 3.6^b^
ACE treated	18.90 ± 3.5	15.83 ± 1.2^c^	13.70 ± 2.1^c^	10.47 ± 1.8^c^>	10.13 ± 2.3^c^

Values are expressed as means (± SEM) of eight to 10 rats for all groups. ^a,b,c^Significant difference (*p* < 0.05) in the same row between various treatments and control group A rats.

## Hexokinase and glucokinase activities

**Fig. 3.**
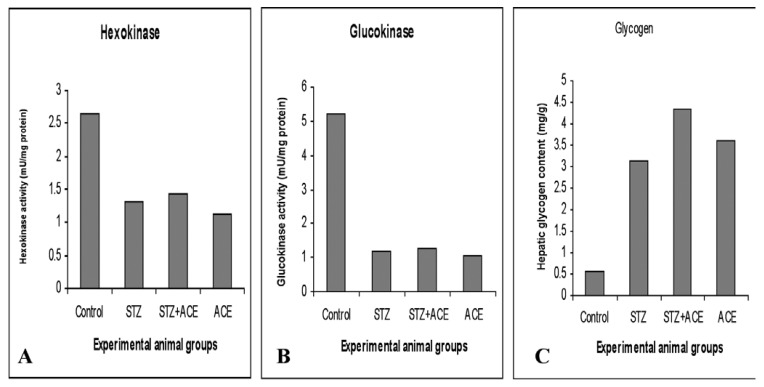
Changes in hepatic (A) hexokinase and (B) glucokinase activities, and (C) hepatic glycogen in liver homogenates after STZ and ACE treatments. Results are expressed as means of eight to 10 observations.

Table 5 shows the effects of ACE on hepatic hexokinase and glucokinase activities. In groups B, C and D treated rats, both hexokinase and glucokinase activities significantly decreased (*p* < 0.05) when compared with the control group (Fig. 3A, 3B).

**Table 5:** Effects Of ACE On HXK And GCK Activities (MU/MG PROTEIN), And Glycogen Content (MG/G) Of STZ-,STZ + ACE- And ACE-Treated Rats

*Parameters*	*Control*	*STZ treated*	*STZ + ACE treated*	*ACE treated*
Liver HXK	2.66 ± 0.3	1.32 ± 0.7^a^	1.42 ± 0.5^a^	1.12 ± 0.3^a^
Liver GCK	5.21 ± 0.4	1.18 ± 0.6^b^	1.25 ± 0.7^b^	1.04 ± 0.1^b^
Liver glycogen	0.57 ± 1.32	3.15 ± 1.56^c^	4.32 ± 0.35^c^	3.59 ± 2.26^c^

Values are expressed as means (± SEM) of eight to 10 rats for all groups. ^a,b,c^Significant difference (*p* < 0.05) in the same row between various treatments and control group A rats.

## Liver glycogen contents

Liver glycogen levels significantly increased (*p* < 0.05) in the diabetic animals treated with STZ, STZ 1 ACE, and ACE when compared with those of the control group rats (Table 5, Fig. 3C). Glycogen levels became markedly elevated in these rats, especially at long-standing diabetic states.

## Discussion

In recent years, phytochemicals from medicinal plants have presented an exciting avenue for the development of new types of therapeutic agents for the management, control and treatment of a plethora of human disorders. This realisation has accelerated the global effort to harness and harvest medicinal plants that bear substantial amounts of phytochemicals with potentially beneficial effects in combating diabetes mellitus and its associated complications. However, only a few of these plants have been subjected to scientific scrutiny, while many still await adequate scientific and biomedical evaluations.^26^

Most reports concerning the toxic effects of herbal medicines are associated with hepatotoxicity, although toxic reports on other cells, tissues, organs and systems, including the kidney, nervous system, blood cells, cardiovascular system and skin, metagenicity and carcinogenicity have also been reported in the medical literature.^27^,^28^ Hepatic impairment resulting from the use of conventional drugs is widely acknowledged, but there is less awareness of the potential hepatoxicity of herbal preparations. Generally, many of the herbal medicines are believed to be harmless and are commonly used for self medication without medical supervision.^29^,^30^

The findings of the present laboratory animal study indicate that aqueous root bark extracts of *A communis* possess hyperglycaemic activity somewhat similar in nature to that of STZ. ACE (≥ 130 mg/kg ip) was also toxic to mice and rats. These findings are quite intriguing because decoctions and infusions of *A communis* root bark are traditionally used by the Yoruba*speaking people of western Nigeria as effective folk remedies for the management, control and treatment of type 2 diabetes mellitus, with no reported adverse effect to date.

The toxicity of the extract observed in the present study confirms our earlier unpublished observations which showed that *A communis* root bark extract was not only lethal to rats at moderate to high doses, but that it also induced hyper-glycaemia in Wistar rats at all dose levels. The toxicity and hyperglycaemia produced by ACE in rats compared to the relative safety and hypoglycaemia produced by the plant extract in human folk medicine may be due to species variation, and/or the different routes through which the extract is metabolised in rat and human livers.

From the data obtained in this study, the animals treated with ACE alone developed acute and severe hyperglycaemia faster than the STZ- or STZ 1 ACE-treated groups of animals. The plant extract also induced moderate body and pancreatic weight losses, with moderate increases in liver weights. Serum and pancreatic insulin levels of the ACE-treated rats were also significantly reduced (*p* < 0.05). Although there was a gradual decrease in the blood glucose concentrations of ACE-treated rats from the third week of our study, the reduction in the blood glucose levels of the animals failed to reach the normoglycaemic levels of the control rats.

Glucokinase is the glucose-sensing enzyme that is responsible for the phosphorylation of the majority of glucose in both the liver and pancreas. GCK binds to and phosphory-lates glucose when glucose levels are higher than normal, thus allowing the liver and pancreas to maintain constant glucose levels.^31^

In the present study, hepatic hexokinase and glucokinase were observed to decrease. This observation is in agreement with the findings of Postic *et al.*^32^ GCK activity was lower in the ACE-, STZ-, and STZ 1 ACE-treated diabetic rats compared with the control rats. The decrease in hepatic GCK could have resulted from hypo-insulinaemia, or decreased synthesis and/or increased degradation of GCK by oxidative stress in diabetes mellitus.^33^ It was also observed that STZ and ACE were capable of inducing liver glycogen accumulation in the treated animals, possibly suggesting decreased glucose utilisation. The high liver glycogen levels obtained in our study could be due to hepatic dysfunction, and/or development of diabetic hepatotoxicity, an observation that is in agreement with the findings of Bischof *et al.*^34^

Although markets for herbal medicines are now booming and evidence for the effectiveness of phytomedicines is growing, trade in herbal medicines is being simultaneously counterbalanced by inadequate regulation. Therefore, standardisation of herbal products, their quality, efficacy and safety, as well as therapeutic risk/benefit associated with the use of herbal medicines need proper evaluation. Based on our findings, we suggest that all plant products should be properly tested *in vitro* and *in vivo* in the laboratory before they are used as remedies in humans.

In conclusion, the findings of the present study indicate that *A communis* root bark aqueous extract induces hyperglycaemia in rats, has deleterious hepatic effects, affects the activities of hexokinase and glucokinase, and increases hepatic glycogen content. Our data also suggest that an ACE-induced diabetic state can cause significant molecular and biochemical changes in the endocrine pancreas.
